# Ruptured Lung Abscess Into the Pleural Cavity: The Significance of Imaging and Medication Compliance

**DOI:** 10.7759/cureus.80077

**Published:** 2025-03-05

**Authors:** Danial Malik, Zaineb Khawar, Ahmed Abbas, Zahra Beizaeipour, Reshma Radhakrishnan, Juilee Dongre, Nader Mahmood

**Affiliations:** 1 Internal Medicine, St. Mary's General Hospital, Passaic, USA; 2 Internal Medicine, Saint Michael's Medical Center, Newark, USA; 3 Pulmonary and Critical Care Medicine, Saint Michael's Medical Center, Newark, USA; 4 Pulmonology and Critical Care Medicine, Saint Michael's Medical Center, Newark, USA; 5 Medical School, Smt. Kashibai Navale Medical College and General Hospital, Pune, IND; 6 Pulmonary Medicine, St. Mary's General Hospital, Passaic, USA

**Keywords:** abscess cavity, bronchopleural fistula, cardiothoracic surgery, empyema of the chest, • lung abscess, necrotizing pneumonia, pulmonary embolism, ruptured lung abscess

## Abstract

A lung abscess is defined as a necrotizing infection with a pus-filled cavity. The infection can be primary in a previously normal lung or secondary to a pre-existing condition such as bronchial obstruction or immunosuppression by HIV or steroid use. Abscesses are more common in men aged 54-74 years, with a history of smoking, alcohol or other sedative use, and immunodeficiency. We present a case of a 65-year-old male with a past medical history of chronic obstructive pulmonary disease (COPD) who presented two months following a necrotizing pneumonia infection and was diagnosed with an acute pulmonary embolism. Due to repeated imaging, an incidental left lower lobe abscess rupture with a bronchopleural fistula was discovered. This case emphasizes the importance of previous chest imaging to establish the disease course, response to therapy, medication compliance, and evaluation for intervention, particularly in underserved populations.

## Introduction

A lung abscess is a necrotizing infection with a pus-filled cavity. This condition may be acute, with symptoms lasting less than six weeks of duration, or chronic. The infection can be primary in a previously normal lung or secondary to a pre-existing condition such as bronchial obstruction or immunosuppression by HIV or steroid use. The mode of spread can be bronchogenic (e.g., aspiration, bronchial obstruction, etc.) or hematogenous (e.g., abdominal sepsis, infective endocarditis, etc.) [1]. 

A bronchopleural fistula is an abnormal connection between a lung abscess and the pleural cavity. Features include an air-fluid level in the pleural space, known as a pyopneumothorax, and air leakage during draining of an empyema. A case report published in the ATS journal discussed that acute pulmonary embolism can also contribute to infarction, abscess formation, and subsequent fistula to an empyema [2].

We present a case of a ruptured lung abscess despite adequate, timely antibiotic therapy, which was detected in a patient diagnosed with bilateral segmental PE.

## Case presentation

This is a case of a 65-year-old male with a past medical history of chronic obstructive pulmonary disease (COPD) on home oxygen, segmental pulmonary embolism with bilateral leg deep vein thrombosis with anticoagulation nonadherence, ischemic cardiomyopathy, and left lung necrotizing pneumonia (two months prior) who received six days of vancomycin and piperacillin-tazobactam, was discharged on three weeks of cefuroxime and metronidazole, and underwent a thoracentesis of 400 mL of thick, blood-tinged exudative fluid per Light’s criteria, with a negative workup for empyema.

He presented to the emergency room at St. Mary’s General Hospital in Passaic, NJ, with three days of exquisite left leg pain, sharp left chest pain with inspiration, progressive shortness of breath, dry cough, and palpitations. On physical examination, he was afebrile, normotensive, had a pulse of 150 beats per minute, exhibited diffuse expiratory wheezes, had diminished air entry on the left lung base with decreased vocal resonance, and had a tender, swollen left calf. The initial laboratory workup was significant for normal white cell count, elevated D dimer of 3 (normal < 0.5 mg/L), and proBNP of 7600 (normal < 900 pg/mL).

**Figure 1:**
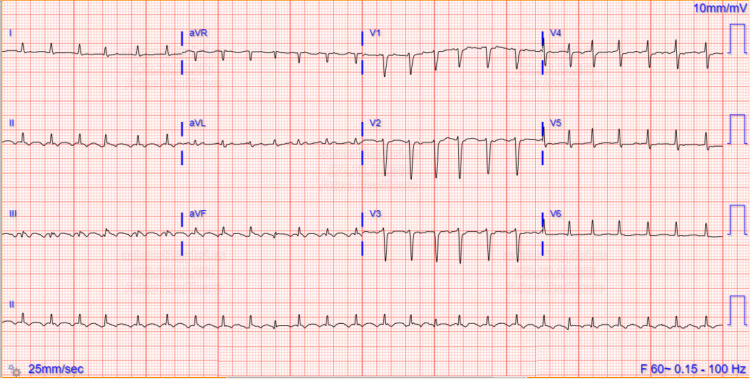
Admission ECG showing new onset atrial flutter with low voltage in limb leads

ECG demonstrated a new onset atrial flutter with low voltage in limb leads, as shown in Figure 1.

**Figure 2:**
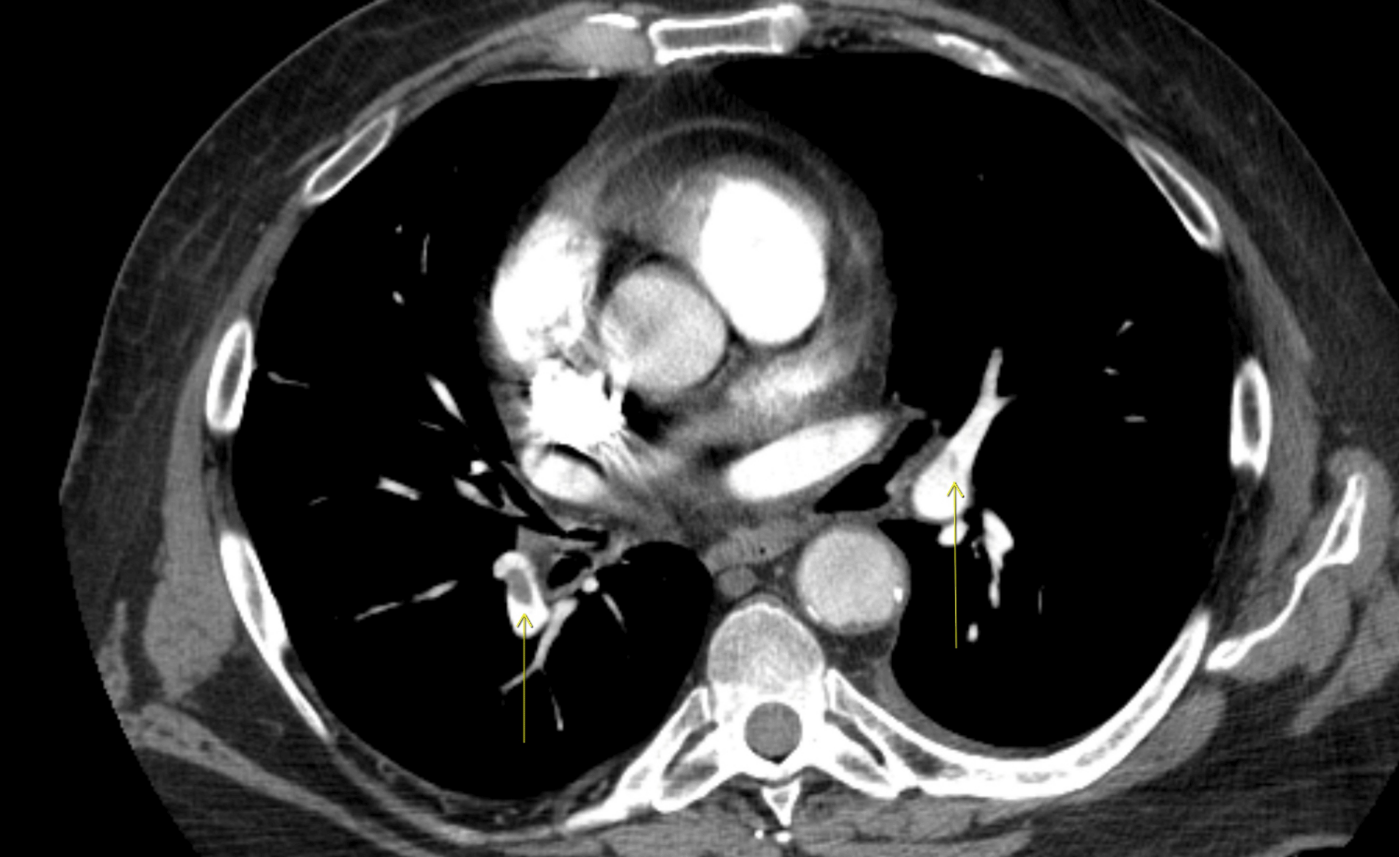
Axial CTA chest mediastinal window showing bilateral lower segmental PE (arrows) CTA, computed tomography with angiography

**Figure 3:**
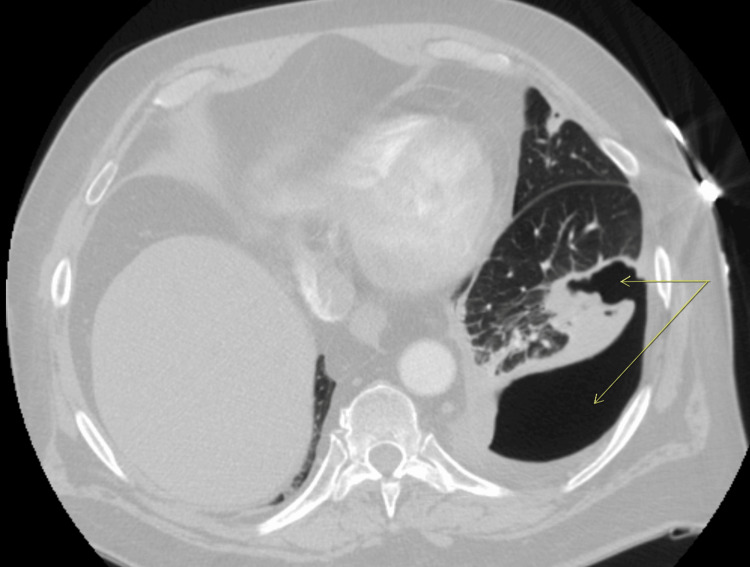
Axial CTA chest lung window showing a left lower lobe thick-walled cavity of 5.6 × 3.9 cm communicating with the adjacent pleura (arrows) CTA, computed tomography with angiography

**Figure 4:**
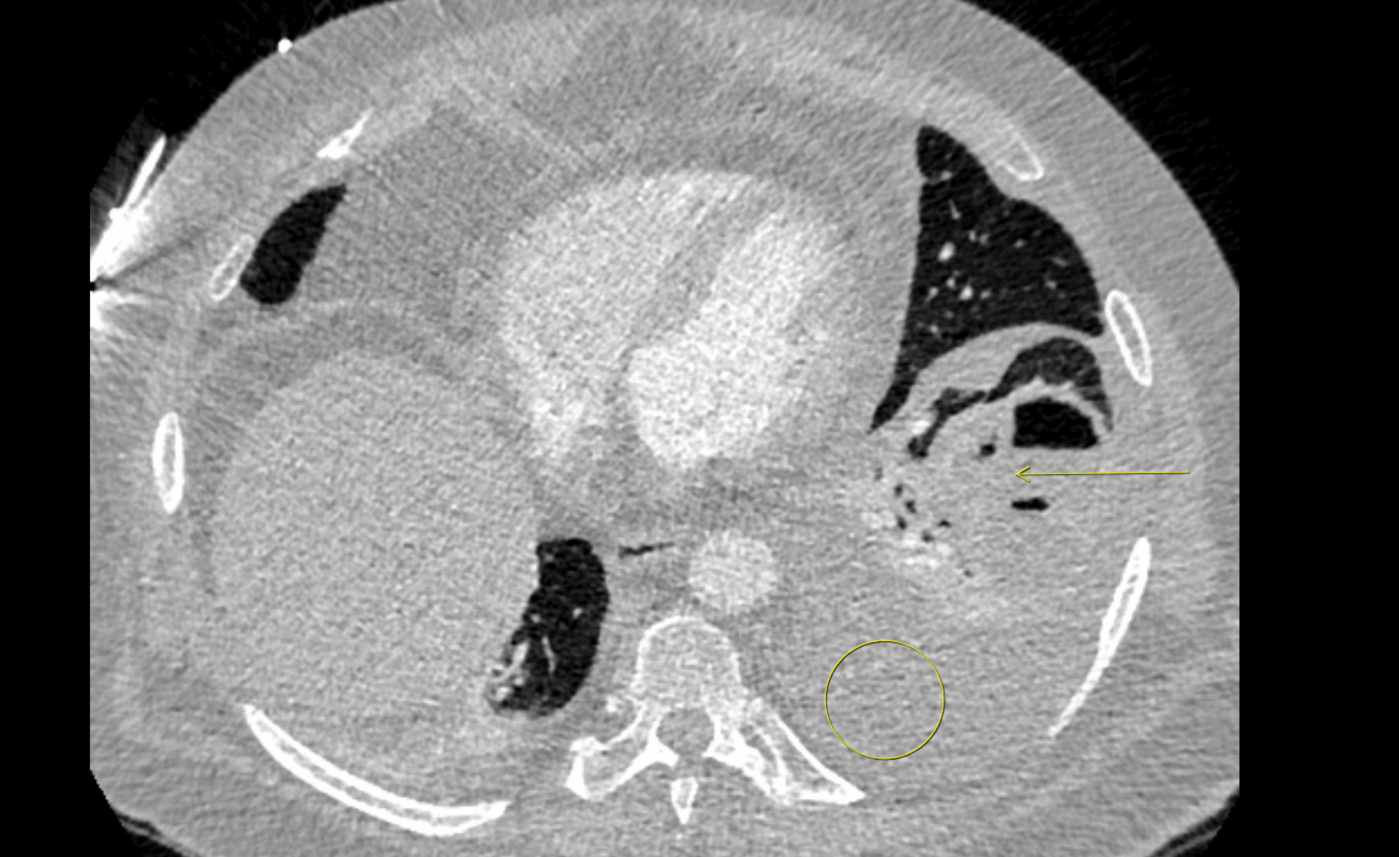
Axial CTA chest lung window two months prior showing left lower lobe consolidation with an abscess cavity (arrow) and moderate left pleural effusion (circle) CTA, computed tomography with angiography

A venous Doppler ultrasound showed acute left leg deep vein thrombosis. A chest computed tomography with angiography (CTA) showed a bilateral lower segmental pulmonary embolism with an incidental left lower lobe thick-walled cavity communicating with the adjacent pleura, as shown in Figures 2, 3, which has progressed from two months prior, as shown in Figure 4.

The patient was admitted to the intensive care unit for an atrial flutter with a rapid ventricular response on a diltiazem drip. Pulmonary and cardiothoracic surgery deemed no intervention for the left lung cavity, given no active infection and the risks deemed to outweigh the benefits. The patient had a transesophageal echo with cardioversion to sinus rhythm. Unfortunately, the patient relapsed into atrial fibrillation. He was managed with a rate control regimen and anticoagulation with counseling on compliance, and he continued to decline continuous positive airway pressure (CPAP) for presumed obstructive sleep apnea. He was discharged to a rehabilitation facility.

## Discussion

Two large retrospective studies were able to identify risk factors regarding the epidemiology of lung abscesses. Pulmonary abscesses were more common in men aged 54-74 years [3,4], with a history of smoking, alcohol or other sedative use, and immunodeficiency. Also, significant literature relates improved dentition to decreased incidence of pulmonary abscess [4]. Pulmonary abscesses in adults are polymicrobial, mainly caused by anaerobic bacteria, such as *Enterobacteriaceae*, *Staphylococcus aureus*, and *Pseudomonas aeruginosa* [3,4].

The initial clinical presentation of a lung abscess cannot be differentiated from pneumonia, with the majority of patients present with cough, pleuritic chest pain, and fever. This pathology is best delineated with a CT chest in terms of chronicity and complications. Acute lung abscess shows a fluid collection with a possible air-fluid level and hazy border owing to active inflammation, whereas chronic lung abscess has an irregular “star-shaped” contour with a well-defined border owing to thick detritus and scar tissue [5].

The majority of lung abscesses respond well to appropriate antibiotics. Surgical drainage is required if the abscess is greater than 6 cm in diameter, complicated by empyema, or if symptoms last more than 12 weeks of appropriate therapy. In case of appropriate, timely antibiotic therapy, immunocompetence, no comorbidities, and young age, the outcome of a lung abscess is generally favorable, where the necrotic tissue will eventually be replaced by scar tissue, and the overall mortality is as low as 2% [5]. 

Owing to his multiple comorbidities, poor dentition, and, most importantly, nonadherence to outpatient antimicrobial therapy, our patient’s lung abscess was complicated by rupture into the pleural cavity, as evidenced by a follow-up CT chest. 

A lung abscess can rupture into the airway, clearing the infection, or it can spread the infection to the healthy lung. Rupture of a pulmonary abscess is an indication for surgical resection and is the therapy choice in up to 10% of cases [5]. Fortunately, our patient wasn’t clinically septic and was deemed appropriate for conservative management as per the surgical team.

## Conclusions

This case emphasizes the clinical challenges of ruptured lung abscess in a patient with multiple comorbidities and the importance of previous chest imaging to establish the disease course, response to therapy, and the need for intervention. It highlights the necessity of compliance with the antibiotic regimen and demonstrates that despite adequate antibiotic therapy, complications may still arise. Finally, it shows the need for outpatient follow-up options that cater to the underserved homeless patient population as a cost-effective measure to prevent future readmissions. This case illustrates the need for further research into the management of lung abscesses and the monitoring of their complications.
